# Bioelectrical Impedance Analysis as a Helpful Tool in Pediatric Obesity Monitoring: A Case Report

**DOI:** 10.3390/reports8010015

**Published:** 2025-01-25

**Authors:** Agata Przytula, Joanna Popiolek-Kalisz

**Affiliations:** 1 Clinical Dietetics Unit, Medical University of Lublin, ul. Chodźki 7, 20-093 Lublin, Poland; 2 Department of Cardiology, Cardinal Wyszynski Hospital in Lublin, al. Krasnicka 100, 20-718 Lublin, Poland

**Keywords:** obesity, children, dietary education, body composition, bioelectrical impedance analysis

## Abstract

**Background and Clinical Significance**: Childhood obesity and its associated complications are an emerging public health problem; thus, non-communicable chronic disease prevention should be implemented as early as possible. On the other hand, obesity management in children is a challenge in terms of achieving fat tissue reduction without any adverse outcomes on overall development. This is why close cooperation with young patients and their parents is crucial for success. Moreover, non-invasive but detailed monitoring guaranties insight in this process’s progress and safety. As obesity is a chronic disease with a tendency for recurrence, further follow-up should also be considered. **Case Presentation**: We present a case of a 10-year-old boy who was referred to a dietitian due to concerns about a diagnosis of obesity and metabolic complications including abnormal lipid profile and liver function. During the dietary consultation, body composition assessment with bioelectrical impedance analysis was conducted, which confirmed obesity. A detailed interview allowed for the identification of improper dietary patterns. The implemented lifestyle education and qualitative diet modifications led to fat mass reduction without any significant muscle loss after just one month. The metabolic profile was also improved. The patient remained under the care of a dietitian for the next 4 years with constant body composition monitoring, which enabled the relevant parties to address if body mass gain was a part of his normal development or if he suffered from obesity recurrence. The patient and his parents benefited from an individualized, patient-centered approach including dietary education, overall lifestyle modification, and detailed body composition monitoring. This way, the patient succeeded in fat content reduction with the constant assessment of the safety of this process. Moreover, the dietary education impacted the whole family’s lifestyle. **Conclusions**: This case emphasizes the role of body composition assessment in children. Obesity and metabolic complications resulting from an improper lifestyle can affect pediatric patients. Bioelectrical impedance analysis is a non-invasive tool that can improve the safety and effectiveness of nutritional interventions and could be included in routine pediatric obesity assessment.

## 1. Introduction and Clinical Significance

Obesity is a chronic metabolic disease and a significant risk factor worldwide. Its occurrence is strongly correlated with the risk of metabolic and cardiovascular diseases, leading to a decrease in quality of life, shortened lifespan, and increased healthcare costs [1]. Unfortunately, obesity is increasingly prevalent among children and youth, which affects the risk of metabolic diseases in future life. Obesity is increasingly observed in children, which is associated with unhealthy lifestyle, including a diet high in saturated fats and a lack of physical activity. It is crucial to implement preventive and therapeutic measures for overweight and obese children and youth to prevent further metabolic complications [2]. Metabolic complications of obesity can include dyslipidemia, elevated blood pressure, and diabetes [3,4,5]. This is already observed in children [6]. This combination is acknowledged as metabolic syndrome, and can lead to atherosclerosis, then to myocardial infarction, stroke, and other cardiovascular complications [7]. This is why it is crucial to diagnose and treat obesity in children to prevent the development of cardiovascular diseases in adulthood.

Various anthropometric markers are available to assess body weight normality. Body mass index (BMI) is used for the basic classification of underweight, normal weight, overweight, and obesity. According to the World Health Organization (WHO), the categories are as follows: underweight for BMI < 18.5 kg/m^2^, normal weight for BMI 18.5–24.9 kg/m^2^, overweight for BMI 25–29.9 kg/m^2^, obesity class I for BMI 30–34.9 kg/m^2^, obesity class II for BMI 35–39.9 kg/m^2^, and obesity class III for BMI ≥ 40 kg/m^2^ [8]. For children, BMI is assessed using percentile charts, which refer the values to age and sex. These charts help determine whether a child’s BMI falls within a healthy range compared to their peers. The percentiles are interpreted as follows: underweight for BMI < 5th percentile, normal weight for BMI from 5th to 85th percentile, overweight for BMI from 85th to 95th percentile, and obesity for BMI > 95th percentile [9].

While BMI is a commonly used method for basic obesity screening and grading, it does not consider muscle mass and fat tissue proportion in the body, so it cannot be used in all populations [10]. People with abdominal fat distribution are at higher risk of metabolic diseases than those with fat tissue in the femoral region. Therefore, BMI may not be the most accurate clinical measure of obesity, as its interpretation depends on the clinical context. That is why the distribution of fat tissue, including waist-to-hip ratio (WHR) calculation, may be a better indicator of metabolic disease risk [11]. It is worth noting that BMI or body mass measurements do not reflect metabolic condition in selected groups of patients, such as athletes, patients with edema, or constitutionally lean patients [11,12]. That is why body composition assessment is so important in terms of obesity diagnosis and monitoring. The gold standard for body composition assessment is the dual-energy X-ray absorptiometry (DEXA) method; however, it requires X-ray application and has numerous limitations such as the cost of the measurement and the size of a device. This is why bioelectrical impedance analysis (BIA) has emerged as an alternative non-invasive method and become popular for assessing body composition in various clinical contexts, including cardiovascular risk. During BIA, a low-intensity alternating electric current (approximately 150 µA) is passed through the body [13]. It is acquired by an even set of electrodes (two, four, or eight) located on the hands and/or feet. The flow of the current between electrodes and the body is performed by holding them with one’s hands or standing on them. Alternatively, self-adhesive electrodes can be attached in dedicated spots. The electrical current is passed through the body, and different tissues are characterized by distinctive conductive properties; thus, the information about the changes in the electrical current coming back to the analyzer serves as the base for body composition prediction, including fat mass (FM) and fat-free mass (FFM), which is calculated by subtracting FM from total body weight. Apart from body composition, BIA parameters provide valuable information about nutritional status at the cellular level [14]. Despite its non-invasive character and safety, BIA is still not widely used in pediatric patients [15,16]. BIA methods have limitations and should not be used in patients with large metal implants (e.g., pacemakers), pregnancy, a fever, or large open wounds or after amputations, as these conditions alter the electrical current flow or body geometry assumed for the body composition parameter calculations.

On the other hand, child obesity is a challenge for dieticians as a caloric deficit should not be introduced as the first-line approach in the course of dietary management in children. However, in the presence of comorbidities, carefully designed behavioral and lifestyle interventions do not exclude the possibility of incorporating a caloric deficit in the management of childhood obesity. Still, body mass reduction could result in muscle mass decline, which is particularly undesirable in children due to their constant development. This is why body composition tracking instead of body mass measurement alone is considered a better approach in the course of obesity management, particularly in children. We present a case of a 10-year-old boy who was referred to a dietician due to obesity and its complications, in whom BIA was successfully used for progress monitoring.

## 2. Case Presentation

### Initial Patient Workup

The 10-year-old boy was referred to a dietitian due to his doctor’s concerns about obesity and its metabolic complications, including abnormal lipid profile and liver function. During the comprehensive dietary consultation, the nutritional interview, health and social assessment, and body composition measurements with BIA were conducted. The patient’s BMI was 31.3 kg/m^2^, which exceeded the 95th percentile. The body composition assessment with BIA (InBody 120 body composition analyzer) revealed that the body fat content was 32.2 kg, which confirmed obesity.

The first body composition analysis was performed 4 h after the last meal, while the subsequent tests were conducted whilst fasting. The examination was carried out in underwear, and the patient was not wearing any jewelry. Before each consultation, the patient was also asked to empty his bladder. Calibration of the device was conducted before each session. The InBody 120 body composition analyzer uses an 8-point tetrapolar touch electrode system, which includes 2 electrodes on the left foot, 2 on the right foot, 2 on the left hand, and 2 on the right hand. It operates at frequencies of 20 and 100 kHz, with a current intensity of 150 µA. The measurement is performed in a barefoot vertical position, with upper and lower limbs separated, and takes 17 s to complete. This device is suitable for individuals aged between 3 and 99 years. The patient did not have any contradictions for BIA measurement.

Before the dietary consultation, the patient’s lipid profile exceeded the normal range for total cholesterol (206 mg/dL), LDL-C (126 mg/dL), non-HDL (160 mg/dL), and triglycerides (172 mg/dL), which indicated atherogenic dyslipidemia. The HDL-C level was 46 mg/dL. Liver function laboratory tests were also performed, and the aspartate aminotransferase (AST) level was 47 U/L, while the alanine aminotransferase (ALT) level was 56 U/L.

The patient was reluctant to engage in physical activity during physical education classes at school, despite being encouraged. There was no known history of familial hypercholesterolemia. The detailed dietary interview allowed for the identification of improper nutritional patterns such as high consumption of sources of saturated fatty acids, such as bacon, sausages, pork, and butter, as well as frequent consumption of salty and sweet snacks. A low consumption of vegetables, fruit, and dairy products was also observed.

Dietary management

The patient was offered a two-week meal plan which incorporated more vegetables and fruit, dairy products, oatmeal, nuts, lentils, and seeds in the everyday diet and reduced the consumption of processed food. The patient was offered a normocaloric diet according to his calculated energy requirements, taking into account the BMR consistent with BIA and the amount of muscle mass (1039 kcal). The applied Physical Activity Level (PAL) factor assumed an increase in the level of physical activity and was considered as 1.7. The final dietary plan provided an average of approximately 1800 kcal daily, 68 g of protein (15% of total daily energy), 60 g of fat (30% of total daily energy), and 248 g of carbohydrates (55% of total daily energy). The plan was based on six meals daily with an interval of 2.5–3 h to adjust the patient’s daily schedule and preferences.

Over the following weeks, the patient’s parents kept a food record, which included the meals and products consumed, physical activity, and information on the amounts of fluids consumed during the day. Based on the records, a significant change in the dietary patterns was noted. As the further step in dietary management, the amount of added sugar was limited. This was achieved by the use of sweeteners such as xylitol and erythritol for the preparation of sweet dishes. Moreover, hummus was used as a spread for sandwiches instead of butter, and salads were added to flour-based meals. Although the patient’s diet still included traditional sweets such as donuts and buns, they were consumed in significantly smaller quantities compared to the previous period.

Results and follow-up

The implemented education regarding lifestyle and modification of the diet, eliminating improper dietary behaviors, led to a significant improvement after one month. A reduction in body weight was observed (BMI 29.02 kg/m^2^), which could be disturbing in a child due to potential concerns about the safety of the introduced interventions in terms of future development. However, it is crucial to highlight that BIA revealed that the decrease in body weight resulted from a reduction in FM (to 25.5 kg), while muscle mass and the total volume of water in the body increased. There are no reference data for FM for the Polish population; however, when using the normal ranges for the U.S. population [17], it was noted that the initial FM percentage was very distant from the 95th percentile and, soon after the intervention, moved closer to the normal range. The eight-point tetrapolar touch electrode system provides the possibility for segmental analysis, including visceral fat assessment (graded by the producer algorithm). In the course of the patient’s follow-up, visceral fat levels also decreased. This way, BIA confirmed the success of the introduced intervention and assured its safety. The metabolic profile of the patient, including liver function and lipid profile, was also improved. The detailed anthropometrical and biochemical results are presented in Table 1.

Next dietary consultations were conducted on a combined basis, both in-person and online. Apart from passively following the dietitian’s recommendations, the patient became involved in preparing meals, and he also suggested recipes he found on the Internet. Moreover, noticeable behavioral changes were observed in other areas of the patient’s lifestyle, as he started playing football and swimming.

This cooperation with the patient and his family has been ongoing since January 2021, with a reduced frequency of consultations. BIA allowed for the monitoring of muscle mass and fat tissue, which was particularly useful as the patient, during such a long follow-up, objectively gained weight as he was in the development stage. BIA assessment enabled us to assess whether his fat and muscle mass were still is a desired proportion. That is why constant BIA monitoring during follow-up enabled us to verify if the behavioral management was still successful.

The patient remained under the care of a dietician for the next 4 years, which allowed for stabilization of laboratory results and a further reduction in fat tissue percentage. Table 2 presents the full history of the patient’s weight and height measurements over a period of 4 years.

**Figure 1:**
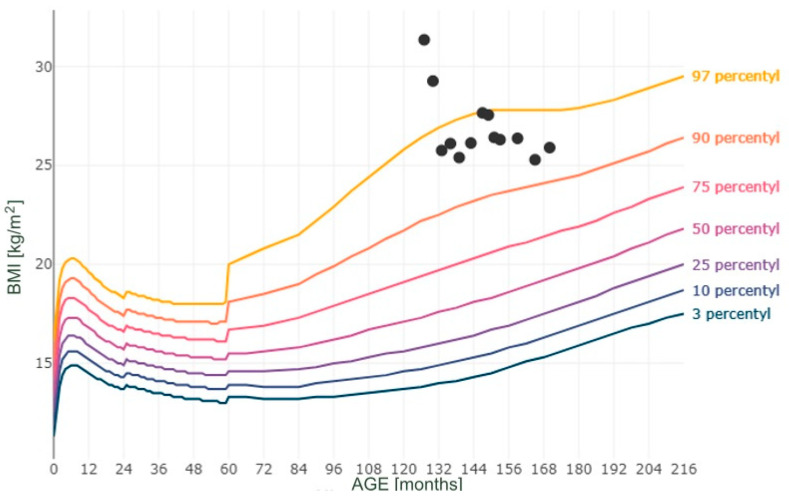
Measurements of BMI plotted as BMI for age on percentile grids.

**Figure 2:**
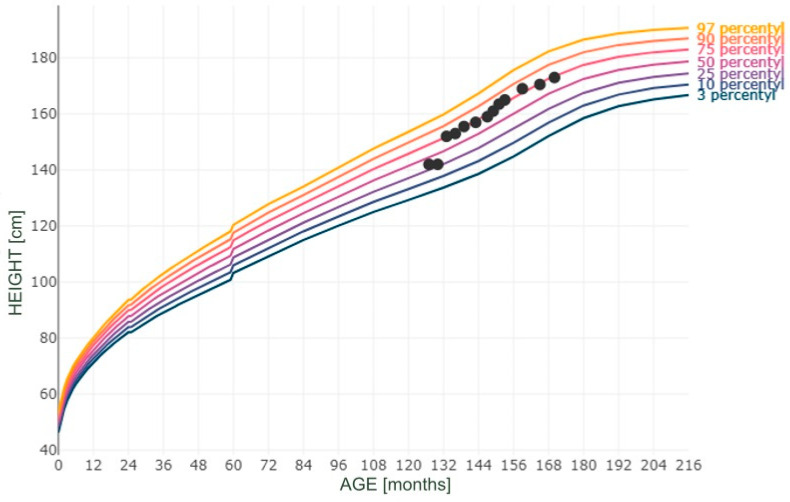
Measurements of height plotted on a height-for-age percentile grid.

**Figure 3:**
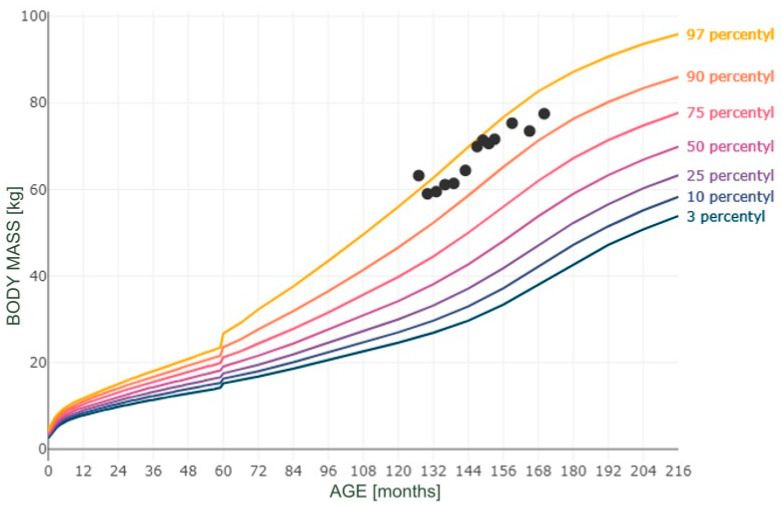
Measurements of body mass plotted on a body mass-for-age percentile grid.

**Figure 4:**
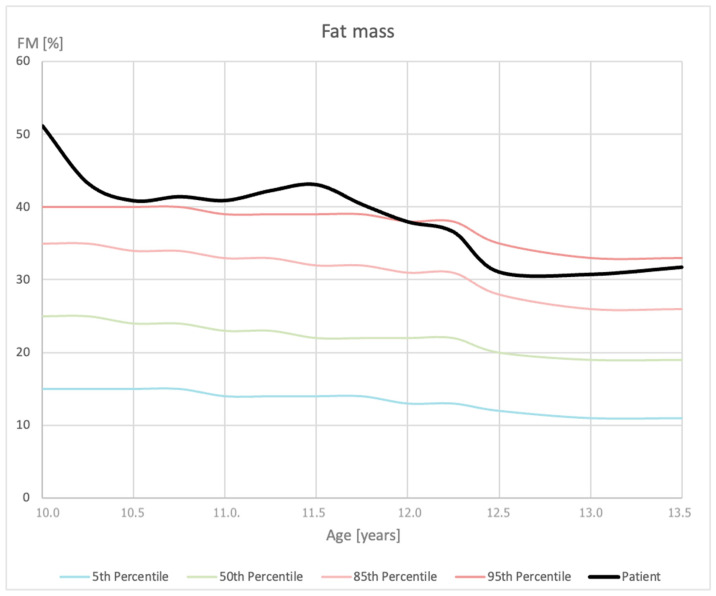
Measurements of fat mass (FM) percentage plotted on a fat percentage-for-age percentile grid.

Moreover, Figures numbered 1 to 3 present the selected measurements plotted on the percentile grids (Figure 1—BMI; Figure 2—height; Figure 3—body mass). The percentiles were based on the results of the OLA and OLAF studies, which serve as the reference values for the Polish population [18]. There are no available validated data for Polish or European populations in terms of FM; however, in Figure 4, we provide the patient’s data compared to the percentile grid for the U.S. population [17].

## 3. Discussion

The development of metabolic disorders is contributed to by sedentary lifestyle and improper dietary habits. That is why initiating lifestyle intervention allows for the reduction in fat tissue levels and lipid parameters. Childhood obesity is a growing health concern worldwide, affecting not only physical health but also the mental and social aspects of a child’s life. The primary factors contributing to childhood obesity include the dietary patterns and lifestyle habits inherited from parents. In previous decades, metabolic disorders such as dyslipidemia in children were mostly the result of genetic diseases, e.g., familial hypercholesterolemia [19,20]. On the other hand, the significant increase in childhood obesity prevalence during recent years led to more frequent cases of childhood dyslipidemia, insulin resistance, and hypertension that were the result of lifestyle behaviors. In the presented case, the observed metabolic disorders were secondary to obesity, which highlight the need for early diagnosis and obesity management in children. It is recognized that body fat amount is associated with lipid profile [21], which also supports the potential of body composition assessment in children with metabolic abnormalities to address whether they can be secondary to excessive FM. In the presented case, body mass and fat reduction resulted in the normalization of the lipid profile, which confirmed this hypothesis.

Obesity management in adults is based on a dietary approach including a caloric deficit and qualitative dietary modifications, e.g., a reduction in saturated fat intake, with an increased consumption of fiber and antioxidants [22,23,24]. In children, due to their developmental stage, a caloric deficit should not be introduced as the first-line approach, so the primary treatment of obesity is based on qualitative dietary changes, which was performed in the presented case. These dietary adjustments aim to promote a healthier lifestyle without the need for restrictive calorie counting, which is crucial for the proper growth and development of children. Introduction of BIA assessment in the patient’s monitoring ensured the safety of the introduced interventions, as the muscle mass and fat content were constantly monitored. Dietary modification and health-promoting lifestyle changes are recommended not only to reduce excessive body mass but also to improve biochemical parameters and achieve specific therapeutic goals through a reduction in body fat [23].

Nutritional therapy is a fundamental aspect of obesity treatment. However, in order to increase the chances of achieving clinical goals, it should not be recommended as the sole method. Instead, it should be combined with other health-promoting lifestyle modifications, such as behavioral and psychological interventions, increased physical activity, and, if indicated, pharmacological or surgical treatment. Moreover, body mass reduction requires a long-term negative energy balance, which cannot be achieved without calorie intake reduction. That is why an increase in energy expenditure from physical activity enables less calorie restriction to achieve similar weight loss results. Additionally, introduction of physical activity combined with proper protein intake prevents muscle tissue loss, which should also be monitored in the course of obesity management, regardless of age. However, maintaining the achieved weight loss is a significant challenge, as continuing a healthy lifestyle can be difficult in the long term, especially if a patient considers it only as a temporary sacrifice. That is why additional education, psychological work-up, and introducing lifestyle changes gradually are the key factors of successful obesity treatment. Apart from dietary and medical consultations, the school environment can serve as a perfect area for lifestyle education targeted at large populations [25]. Physical education classes performed within the education program are a way to implement structured activity; however, unstructured outdoor play at school provides children with additional opportunities to take part in moderate-to-vigorous physical activity [25]. This policy can positively impact body fat in children and prevent childhood obesity.

As already mentioned, monitoring obesity treatment is another challenge, as the implemented actions are aimed to reduce body fat. Unfortunately, body mass reduction can also lead to muscle mass decrease, which is an unfavorable outcome, particularly in children. That is why obesity management in children requires particular attention. Although BMI is a cheap and simple measure of body mass, it does not indicate the distribution of fat tissue in the body, nor the amount of muscle mass and fat tissue. BMI can be an effective indicator for children with a standard fat-to-muscle ratio, but it can be misleading for children with a higher proportion of muscle or fat. BMI also does not always accurately reflect age-related changes. As age increases, the proportion of fat tissue rises while muscle mass decreases, but changes in height, weight, and BMI may not reflect these changes in fat and muscle tissue [26]. Additionally, the relationship between BMI and body fat percentage is not linear and differs between sexes [27].

The application of BMI alone can lead to significant misclassifications, such as categorizing overweight or obese children as having a normal weight and vice versa. Measuring total body fat is considered a better approach because it provides estimates of lean mass and FM. Therefore, the measured body fat percentage can serve as a criterion for assessing overweight and obesity among adolescents [28]. Moreover, it was reported that treatment-naïve transgender male adolescents have an imbalance between muscle and adipose tissue, which places them at increased susceptibility for metabolic syndrome components, even prior to hormonal treatment, which is another example of the importance of body composition acknowledgement in a patient’s assessment [29]. BIA can help assess nutritional status and monitor the impact of dietary and lifestyle changes on body composition. Moreover, it can serve a purpose for fluid balance assessment [30].

As already mentioned, young patients who are still in the development period should be particularly monitored for muscle tissue loss. As highlighted, an accurate and efficient body composition assessment is essential for diagnosing and monitoring childhood obesity in clinical and research settings [31]. That is why BIA is an exceptionally useful tool for this purpose [29]. The gold standard for body composition assessment is dual-energy X-ray absorptiometry; however, due to high-energy electromagnetic radiation, it is contradicted in children, which elevates the role of BIA in pediatric patients, as recent BIA devices offer precise measurements of the percent of body fat [21]. Recent studies indicated that, compared to dual-energy X-ray absorptiometry, BIA is a valuable tool for monitoring body composition in children with obesity, particularly in longitudinal observations [31,32]. In the presented case, overall body mass values presented an increasing trend. However, when body composition information was considered, we observed an increase in muscle mass and a decrease in fat tissue. There was a noticeable downward trend in visceral fat levels observed as well. Selected studies report that compared to dual-energy X-ray absorptiometry, BIA tends to overestimate FFM and underestimate FM in children [31,33], but without significance [32,34], but performing BIA measurements in the same conditions can improve its accuracy, particularly during follow-up, which was presented in this case. On the other hand, the lack of reference data for the European population makes this method still underused. This case highlights the need for creating percentile grids for body composition parameters in the European pediatric population.

The presented case demonstrates the importance of muscle mass and fat tissue quantity assessment rather than body mass measurements alone in the course of obesity treatment. It also highlights the importance of nutritional education and the dietitian’s work with parents and children to prevent cardiovascular diseases.

This case also underlines the importance of long-term and individualized cooperation with obese patients to maintain the achieved goals. The patient reported feeling more confident and motivated as noticeable changes in body composition and improved laboratory results were observed. The incorporation of BIA provided additional reassurance for parents of the treatment process’s safety. Additionally, the personalized approach, which included dietary education and meal adjustments tailored to the patient’s preferences, provided a sense of autonomy and increased the patient’s engagement level. As the patient’s parents were also included in the education process, the changes positively impacted the whole family’s lifestyle. Altogether, this patient-centered approach resulted in gaining patient trust and providing comfort in the course of the obesity treatment.

Despite its practical value, this case report has its limitations. The presented conclusions are based on one case; thus, larger studies are needed for generalizing findings. Moreover, the case is described from a dietician’s perspective, so additional medical tests available only on doctors’ orders would be valuable to increase the level of presentation.

## 4. Conclusions

Obesity and metabolic disorders resulting from an improper lifestyle already affect pediatric patients. Obesity management in children is a particular challenge due to potential risks of impacting development. This case showed that including body composition analysis measurements with BIA is useful in obesity treatment monitoring to ensure its safety and efficacy, particularly in children.

## Figures and Tables

**Table 1:** Modifications in anthropometric and biochemical markers through time.

	Before a Dietary Consultation	One Month After Introducing Lifestyle Changes	March 2023	August 2024	Units
Body mass	63.2	59.0	71.6	77.5	kg
Skeletal muscle mass	16.3	17.7	24.8	29.3	kg
Fat mass	32.2	25.5	26.2	24.6	kg
Total cholesterol	206.0	169.0	162.0	144.0	mg/dL
LDL-C	126.0	102.0	89.4	81.4	mg/dL
non-HDL	160.0	123.0	110.0	96.0	mg/dL
Triglycerides	172.0	104.0	103.0	73.0	mg/dL
HDL-C	46.0	46.0	52.0	48.0	mg/dL
AST	47.0	35.0	No measurement	26.0	U/L
ALT	56.0	32.0	No measurement	18.0	U/L

**Table 2:** The full history of the anthropometric and body composition measurements.

	Date [yy/mm/dd]	Height [cm]	Body Mass [kg]	Muscle Mass [kg]	Fat Mass [kg]	BMI [kg/m²]	Fat Level Visceral [AU]	BMR [kcal]
1.	21.01.14	142.0	63.2	16.3	32.3	31.34	17	1039
2.	21.04.17	142.0	59.0	17.7	25.5	29.26	14	1092
3.	21.07.10	152.0	59.5	18.8	24.3	25.75	13	1131
4.	21.10.16	153.0	61.1	19.1	25.3	26.10	13	1143
5.	22.01.08	155.5	61.4	19.4	25.1	25.39	13	1154
6.	22.04.30	157.0	64.4	20.0	27.2	26.13	14	1174
7.	22.09.17	159.0	69.9	21.5	30.1	27.65	16	1228
8.	22.11.19	161.0	71.4	23.1	28.8	27.55	14	1290
9.	23.01.28	163.5	70.6	23.8	26.8	26.41	13	1315
10.	23.03.11	165.0	71.6	24.8	26.2	26.30	12	1350
11.	23.09.02	169.0	75.3	28.7	23.4	26.36	10	1491
12.	24.03.09	170.5	73.5	28.1	22.6	25.28	9	1470
13.	24.08.27	173.0	77.5	29.3	24.6	25.90	10	1512

## Data Availability

Data regarding this case are available upon reasonable request to the corresponding author due to privacy concern.
